# COVID-19 mRNA vaccine and antibody response in lactating women: a prospective cohort study

**DOI:** 10.1186/s12884-021-04051-6

**Published:** 2021-09-17

**Authors:** Nadia Charepe, Juliana Gonçalves, A. Margarida Juliano, David G. Lopes, Helena Canhão, Helena Soares, e Fátima Serrano

**Affiliations:** 1 Centro Hospitalar Universitário de Lisboa Central (CHULC), Lisboa, Portugal; 2 Comprehensive Health Research Centre, NOVA Medical School, Universidade NOVA de Lisboa, Lisboa, Portugal; 3 Human Immunobiology and Pathogenesis Laboratory, Lisbon, Portugal; 4 CEDOC-Chronic Diseases Research Center NOVA Medical School | Faculdade de Ciências Médicas, NOVA University of Lisbon, Lisbon, Portugal; 5 EpiDoC Unit, CEDOC, NOVA Medical School, Universidade NOVA de Lisboa, Lisboa, Portugal

**Keywords:** Breastfeeding, Covid-19, mRNA vaccination, Antibodies

## Abstract

**Background:**

Immunological protection via breastfeeding is well known. The immunological profile of human milk changes during lactation. No clinical trials have been conducted in lactating women with the newest mRNA vaccines against SARS- CoV-2. A Few studies have shown the presence of antibodies in breastmilk after vaccination. The aim of this work is to study possible antibodies transfer via breastmilk and also the immunological characteristics of lactating women compared to non-lactating women, after using the BNT162b2 Pfizer vaccine.

**Methods:**

This is a prospective cohort study with a convenience homogenous sample of 24 healthcare workers (14 lactating and 10 non-lactating women) enrolled at the time of COVID-19 vaccination. Clinical data was registered in a questionnaire. Titers of SARS-CoV-2 spike IgG, IgA and IgM were quantified in post vaccination blood and human milk. Antibody quantification was performed by an in-house ELISA to SARS-CoV-2 trimeric spike protein.

**Results:**

All women showed immunity after vaccination with positive antibodies for IgM, IgA and IgG antibodies. The dominant serum antibody response was IgG. Modest levels of antibodies in breastmilk of lactating mothers were observed in this study, especially IgG in 42.9%. There was a moderate association between higher titers of IgG and a longer duration of breastfeeding (R= 0.55, p=0.041).

**Conclusions:**

Evidence of antibody transfer in human milk after COVID-19 vaccination is scarce. The presence of antibodies in human milk is reported, but immunization through breastfeeding is still to be established.

## Background

In the first months of life, neonates are at greater risk of infections, due to their immature immune system and breastfeeding will boost immunological responses [1]. Additionally, breastfeeding is proven to be effective against acute and prolonged infections, and has an influence on infant immune response after mother’s vaccine immunization [2]. Secretory IgA (SIgA) represents 90% of the antibodies in human milk, followed by IgM and IgG antibodies [1]. For this reason, and for its biological properties, SIgA is very important, as it is essential in defending mucous membranes [1]. Nevertheless, specific characteristics of lactating mothers may influence the kinetics of human milk antibodies due to the differences of previous infections (time since preexisting disease immunity), age, genetic factors, and individual immune response [3].

The arrival of COVID-19 vaccines, specifically mRNA vaccines against SARS-CoV-2 such as BNT162b2 Pfizer, has raised the question whether they are safe for use in the pregnant population and lactating women, because no clinical trials were conducted on these groups. In the general population, mainly participants without previous SARS-CoV-2 infection, there was a 90% efficacy at preventing severe symptoms after two doses were administered [4]. The academy of Breastfeeding Medicine does not recommend cessation of breastfeeding for individuals who are vaccinated against COVID-19 [5]. The American college of obstetricians and gynecologists recommend that COVID-19 vaccines should be offered to lactating individuals as to non-lactating individuals [6]. The potential risks and benefits for these breastfed babies are still to be determined. Taking into account that vaccination during pregnancy against other viruses, such as influenza, was related to specific active antibody production throughout lactation, the same may happen in mRNA vaccines [7].

Gray *et al*, confirmed that the COVID-19 mRNA vaccines result in comparable humoral immune responses in lactating women to those observed in non-pregnant populations after studying 31 lactating women in a prospective cohort study. Antibodies and especially SARS-CoV-2-specific IgG were found in breastmilk [8].

The objectives of this study were to evaluate the serological profile of lactating women compared to non-lactating women, after immunization with the BNT162b2 Pfizer vaccine, in a cohort of healthcare workers, and study antibody transfer via breastmilk.

### Materials and methods

This was a prospective cohort study undertaken between 27 December 2020 and 19 February 2021, with a convenience homogeneous sample of 24 healthcare workers (14 lactating and 10 non-lactating women) enrolled at the time of COVID-19 vaccination with BNT162b2 Pfizer/BioNTech after advertising the study.

Eligible participants were: 1) lactating and non-lactating women who underwent vaccination with BNT162b2 Pfizer; 2) older than 18 years old and able to provide informed consent.

Demographic data, date and type of delivery, breastfeeding details, timing of COVID-19 vaccine doses, post-vaccination symptoms were assessed using a written questionnaire.

This study was reviewed and approved by the local ethics committee (NOVA Medical School) as per principles embodied in the Declaration of Helsinki. Written informed consent was given by all participants.

### Samples collection

A blood sample was collected by venipuncture from all participants one to three weeks after the first and second dose of vaccine administration (accordingly with participants availability). Breastmilk from lactating women was collected on the same days of blood collection (after the first and second dose) and collected with breast pump into sterile containers, being the volume around 100-200 ml (Table 1).

**Table 1:** Elapsed time (days) after the 1^st^ and 2^nd^ dose

ID Lactating	1st dose	2nd dose	ID Non-Lactating	1st dose	2nd dose
1	10	10	15	22	10
2	8	8	16	22	16
3	8	10	17	22	10
4	10	10	18	22	10
5	13	13	19	22	10
6	9	7	20	22	10
7	8	9	21	22	10
8	8	9	22	23	11
9	7	10	23	23	11
10	12	12	24	23	11
11	10	11	-	-	-
12	13	10	-	-	-
13	8	7	-	-	-
14	16	9	-	-	-
**Mean (SD)**	9.5 (2.6)	10 (1.7)	**Mean (SD)**	22.0 (0.5)	10.0 (1.9)

SD Standard Deviation

### Antibody quantification: Plasma and skim milk isolation

Blood was diluted in PBS 1x (VWR), layered on top of biocoll (biowest) and centrifuged at 1200xg for 30 min without a break. Plasma was collected to cryotubes and stored at -80°C ultra-low freezer until subsequent analysis.

Skim milk was stored in -20°C freezer until further analysis. Both biospecimens were immediately processed and centrifuged at 3000xg for 30 min at room temperature. ELISA assay was performed based on the protocol [9] and modified as described in Gonçalves J *et al* [10].

Briefly, 96 well plates (Nunc) were coated with 50 μl of trimeric spike protein at 0.5 μg/mL and incubated overnight at 4°C. On the following day, the plate was washed three times with 0.1% PBS/Tween20 (PBST) using an automatic plate washer (ThermoScientific). Plates were blocked with 3% of bovine serum albumin (BSA) diluted in 0.05% PBS/T and incubated for 1 hour at room temperature. Samples were diluted using 3-fold dilutions series, starting at 1:50 and ending at 1:10,9350 in 1% BSA-PBST/T and incubated for 1 hour at room temperature. As previously, Plates were washed three times and goat anti-human IgA/IgG/IgM-HRP secondary antibodies (abcam, ab97225/ab97215/ab97205) were added at 1:25,000 and incubated for 30 minutes at room temperature. Plates were washed three times and incubated for around 7min with 50 μl of TMB substrate (BioLegend). The reaction was stopped with 25 μl of 1M phosphoric acid (Sigma) and read at 450nm on a plate reader (BioTek).

Each plate contained 6 calibrators samples from two high-, two medium-, and two low- antibody producer from adult individuals collected at Fernando Fonseca Hospital that were confirmed positive for SARS-CoV-2 by RT-PCR from nasopharyngeal and/or oropharyngeal swabs in a laboratory certified by the Portuguese National Health Authorities [10]. For negative control we used pre-pandemic plasma samples obtained from healthy donors collected prior to July 2019.

The endpoint titer was defined as the last dilution before the absorbance dropped below OD_450_ of 0.15. For samples that exceeded an OD_450_ of 0.15 at the last dilution (1:10,9350) the end-point titer was determined by interpolation [9]. Positive immune response (IgG, IgA and IgM) was considered when ≥ 150 UI/mL. We calculated IgG and IgA endpoint titers of SARS-CoV-2 positive individuals by serial 3-fold dilution and classified end-point titers of 1:150 as low, 1:450 as moderate, and ≥1:1.350 as high antibody producers, as previously done [9].

### Statistical analyses

Continuous variables were expressed as means, standard deviation and range, whilst categorical variables were shown as absolute frequencies and percentages. The study used IBM SPSS® version 23 and RStudio software [11] for statistical analysis. Correlation analyses were performed using the Spearman coefficient. Experimental and control groups antibody levels were compared using the Wilcoxon-Mann-Whitney non-parametric test. Statistical significance was defined as p <0.05.

## Results

Participant’s characteristics are presented in Table 2.

**Table 2:** Participant’s data

Characteristics	Lactating women (n= 14)	Non-lactating women (n= 10)
**Maternal data**		
Age Median ±SD	33.7 ± 4.95 years	34.5 ±10.6 years
(minimum, maximum) years	26-44	26-62
Caucasian n (%)	14 (100%)	10 (100%)
Smoking habits n (%)	1 (7.1%)	1 (10%)
Comorbidities n (%)		
No Comorbidities	8 (57.1%)	6 (60 %)
Hypertension	1 (7.1%)	1 (10%)
Asthma	2 (14.3%)	-
Dyslipidaemia	1 (7.1%)	1 (10%)
Thyroid disorders	1 (7.1%)	1 (10%)
Beta thalassemia	1 (7.1%)	-
Hodgkin lymphoma	-	1 (10%)
Side effects post vaccination n (%)		
Myalgia	8 (57%)	6 (60%)
Headache	6 (42.9)	5 (50%)
Local pain	5 (35.7%)	4 (40%)
Nausea	2 (14.3%)	2 (20%)
Fever	1 (7.1%)	2 (20%)
Photophobia	1 (7.1%)	2 (20%)
Tiredness	-	3 (30%)
Arthralgia	1 (7.1%)	2 (20%)
Arm numbness	1 (7.1%)	-
**Delivery data**		NA
GA at delivery Median ±SD (Range)	38.5 ± 1.7 weeks (range 34-41)	
Mode of Delivery n (%)		
Caesarean	5 (35.7%)	
Vaginal	9 (64.3%)	
**Infant and Breastfeeding data**		NA
Birthweight (g) Median ±SD	3263 ± 383 (2460-3800)	
Percentile at the time of vaccination	65.5±17 (range 50-97)	
Breastfeeding duration n (%)		
≤ 2 - 12 months	6 (42.85%)	
≥ 12 - 24 months	8 (57.14%)	
Exclusive breastfeeding	2 (14.28%)	

*GA* Gestational age, *NA* Not applicable

There were no differences between lactating and non-lactating women in terms of age, smoking habits or comorbidities. None of the participants reported a prior SARS-CoV-2 infection. There was no difference in post vaccination side effects when comparing lactating versus non lactating women (*p*-value = 0.618). The most commonly reported side effect was myalgia.

Delivery occurred at term in 92.8% (n=13) of the lactating women and 64.3% (n=9) had a vaginal delivery. Most lactating women had breastfed over a time period of 12-24 months at the time of the sample collection. Exclusive breastfeeding was registered in 2 cases. The weight of all infants at the time of the study was ≥ 50^th^ percentile.

**Fig. 1:**
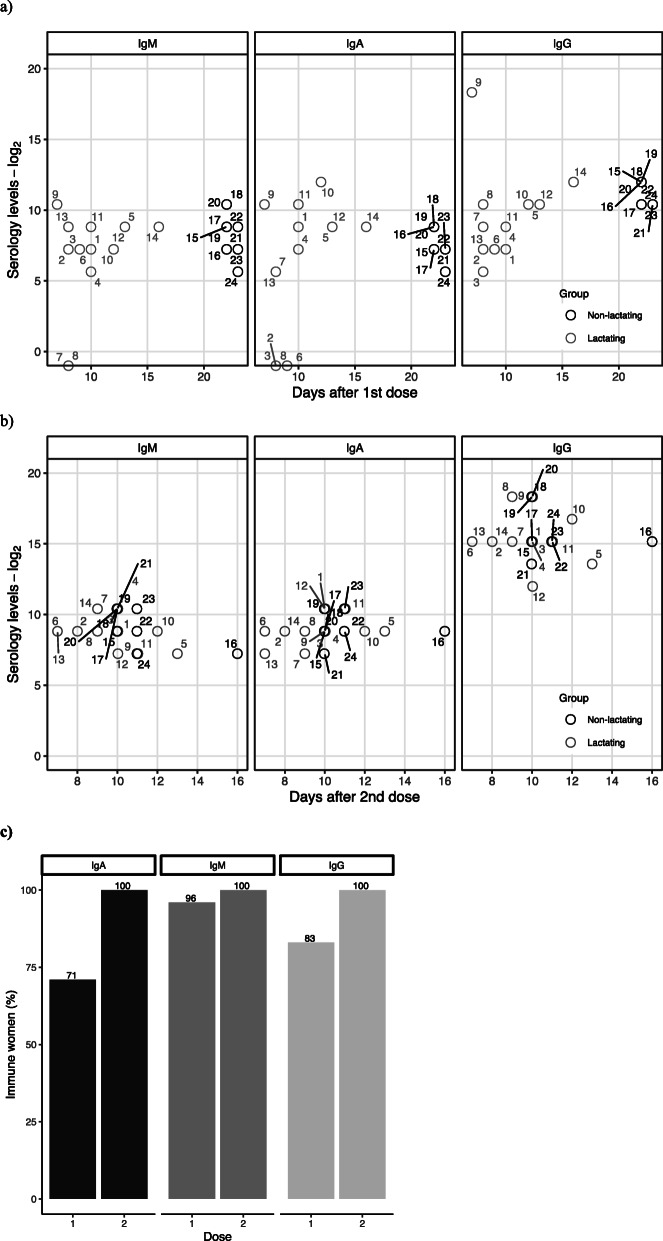
Distribution of titers over elapsed time (days) after the first dose **a** and second dose **b** of the BNT162b2 Pfizer vaccine for lactating (n=14) and non-lactating (n=10) women. **c** Percentage of women with vaccine-induced antibodies (antibody titers ≥150) c)

All women have shown immunity after vaccination with positive serum antibodies after the second dose (Fig. 1). The dominant serum antibody response was IgG, showing high levels in both lactating and non-lactating women. (Table 3) However, after the 1^st^ dose there were higher levels of serum IgG antibodies in the non-lactating group (W = 112.5, *p*-value = 0.00531).

**Table 3:** Antibody titers among lactating and non-lactating women

Antibodies titers	Lactating women (n=14)		Non-lactating women (n=10)	
Blood (Mean)	1^**st**^ dose (UI/mL)	2^**nd**^ dose (UI/mL)	1^**st**^ dose (UI/mL)	2^**nd**^ dose (UI/mL)
**IgG**	24250	79264	2700	121500
**IgA**	880 (4 ND)	600 (2 ND)	260	690
**IgM**	366	621	530	930
**Milk (titer range 150-1350)**				
**IgG**	1 (7.1% )	6 (42.9 %)	NA	NA
**IgA**	5 (35.7% )	3 (21.4%)	NA	NA
**IgM**	ND	ND	NA	NA

*ND* not detected, *NA* not applicable

**Fig. 2:**
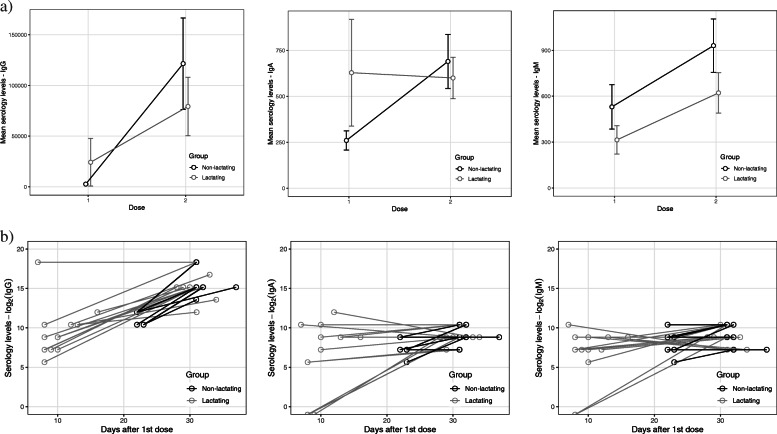
Mean blood serology levels in lactating (n=14) and non-lactating (n=10) women after the first and second dose of BNT162b2 Pfizer vaccine **a**) Mean IgG, IgA, IgM isotypes (log2) over elapsed time from 1^st^ dose **b**) Full sample n=24

Regarding lactating women, serum IgM and IgG isotypes increased after the 2^nd^ dose. IgA levels reduced slightly after the 2^nd^ dose, though there was great variation in individual values (Fig. 2).

The presence of antibodies in breastmilk was detected after vaccine administration. IgG was present in 7.1% (1/14) after the 1^st^ dose and increased after the 2^nd^ dose to 42.9 % (6/14). IgA response was present in 35.7% (5/14) of milk samples after the 1^st^ dose, but showed a reduction to 21.4% (3/14) after the 2^nd^ dose. No IgM response was observed following the prime or boost. (Table 2)

**Fig. 3:**
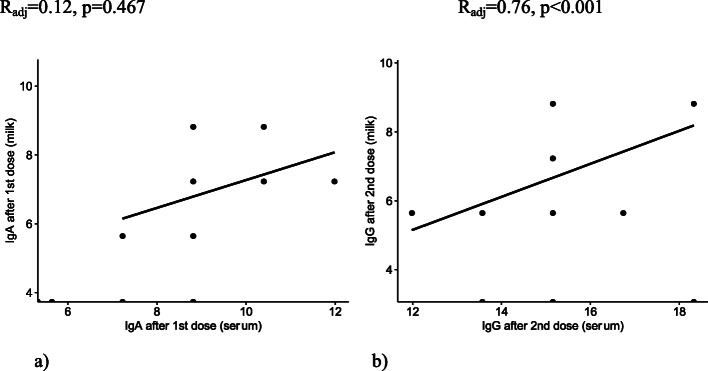
The Spearman coefficient of correlation (log_2 antibodies) between IgA (1^st^ dose) in milk and serum **a**, and IgG (2^nd^ dose) in milk and serum **b**, adjusted for maternal age and time after the vaccine dose (days)

When comparing IgA (1^st^ dose) and IgG (2^nd^ dose) levels between matched serum/milk, there was a tendency for an upward curve and a statistically significant association between IgG (2^nd^ dose) serum/milk when adjusting for maternal age and days after dose administration (Fig. 3).

**Fig. 4:**
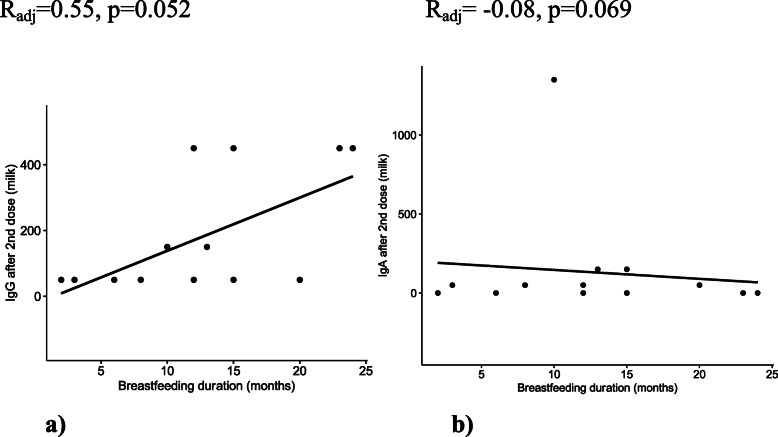
**a** Correlation between breastfeeding duration and milk IgG after 2^nd^ dose. **b** Correlation between breastfeeding duration and milk IgA after 2^nd^ dose

We analyzed the relationship of breastfeeding duration and IgG/IgA response in the milk. Moderate positive correlation between the two variables (duration of breastfeeding and milk IgG after 2^nd^ dose) was found (R= 0.55, *p*=0.041). Thus, higher titers of IgG are associated with longer breastfeeding time (Fig. 4a) However, when adjusted for maternal age and time since the 2^nd^ dose, this correlation was no longer statistically significant. Additionally, the distribution of values for a) does not appear to be linear.

This is likely owing to the small number of observations which happened to have the same IgG titer values. There was a non-significant negative association between IgA (2^nd^ dose) and duration of breastfeeding (Fig. 4b).

All women kept breastfeeding post vaccination. Only one infant showed different behaviour after maternal vaccination (somnolence). There was a case of tandem breastfeeding (brothers) not related to increase transfer of antibodies to the milk.

## Discussion

Pregnant and lactating women were excluded from initial clinical trials for anti-SARS-CoV-2 vaccination, and many concerns, especially safety issues, arose related to vaccination in this specific population. To our knowledge, only a few studies have addressed this topic [12–15]. To date, there are no robust studies regarding the safety and efficiency of vaccination in lactating women. In the study of Golan et al, mRNA from anti-COVID vaccines was not detected in human breast milk, this strengthens the recommendation of maintaining breastfeeding after inoculation [16]. In a recent study of Friedman et al, there is evidence of a rapid production of vaccine-specific antibodies, both IgA and IgG and neutralizing capacity was proven [17]. Moreover, information is needed regarding antibody transfer to breastmilk and the ability of the infant to receive immunity via breastfeeding, response that is well established in other vaccines during pregnancy such as Influenza and Pertussis [18]. Due to the lack of information, our study aims to add scientific information, allowing future studies to develop.

In our cohort, the presence of serum antibodies after vaccine administration was documented in all women. In a non-infected population, vaccination with mRNA vaccines elicited spike antigen-specific IgA with similar kinetics of induction as IgG, although the levels of spike antigen-specific IgA decreased significantly over time [13]. We also documented a significant increase in IgG response after the second dose. On the other hand, IgA response decreased after the second dose. These results are comparable to previous studies [12, 13].

An increase in antibody titers in non-lactating women comparing to lactating women in our study could be explained by longer time till measurement of titers in non-lactating group, allowing antibodies to reach their full peak.Regarding human milk, there was an IgA dominant response in women that were infected with SARS-CoV-2 [19]. A modest response of antibodies in human milk with an IgG dominant response was demonstrated. IgA titers, although present, decreased by the time of the second dose and no IgM was detected. When comparing titers of antibodies in serum and milk at the same time point, a similar response between them was noticed, although, lower levels of antibodies were present in the milk.

The dominant IgG response in blood and milk after COVID-19 vaccination can be related to an exposition of viral spike protein through an intramuscular injection. Contrarily, greater IgA response is registered in natural infection, probably due to the fact that infection occurs in mucosal tissues where IgA response plays an important role. The mean value of IgG titers after the first dose (Fig. 2) for non-lactating women is higher than the lactating group due to the inclusion of an outlier value. This individual was maintained so not to reduce the sample size further. The fact that after the 2^nd^ dose, other individuals reached the same magnitude of titers indicates that the value is not itself senseless, but the sample size is small to encompass such variations. The comparison of the values distribution in both groups after the first dose showed that, in fact, non-lactating women had significantly higher levels of IgG antibodies.

During lactation, the immunological profile of human milk changes over time. SIgA is very high in colostrum, decreasing and remaining stable until 1 year of lactation. After this period, SIgA and IgG show an upward trend and IgM remains stable, supporting the importance of breastfeeding after one year of age for its immunological protection. These results might be associated with the higher production of antibodies in lactation over 12 months already demonstrated in other studies [20]. A correlation was also found between higher titter of antibodies in human milk in women that were breastfeeding for a longer time, although not significant after adjusting for maternal age and days after vaccine administration.

Further research is needed for a better knowledge of longevity of these antibodies in breastmilk and if they are transferred efficiently to the infant.

### Strengths and limitations

One of the strengths of this study is related to the novelty of the topic, since little is known about COVID-19 vaccine immunization and breastfeeding. As breastfeeding brings so many advantages to the newborn and infant, it was considered of extreme importance, studies regarding vaccine and immune response in this population. This is the first study to compare the duration of breastfeeding with higher levels of antibodies in maternal milk.

In terms of limitations, previous infection to SARS-CoV-2 could not be ruled out, as no antibody testing was performed before vaccination. However, participant self-reported that they were not previously infected and as they are health workers they are routinely tested. The small sample size limited statistical inference.

The increased levels of antibodies in non-lactating women after first dose was higher than in the lactating group probably due to a longer time until sample collection.

More than 90% of the human population is seropositive for at least three Human Coronavirus; therefore cross reactivity can also be an issue as it is speculated that this reflects T cell memory to circulating ‘common cold’ coronaviruses [21].

## Conclusion

Currently, the evidence of antibodies transfer in human milk after COVID-19 vaccination is scarce. This is a first insight into vaccination and lactation, highlighting the questions that need to be answered. Also, the belief is, as per the findings, that long-term lactating women may show different serological milk responses after vaccination. Clinical trials are immediately required in this specific population in order to address scientific based recommendations.

## Data Availability

The datasets used and/or analyzed during the current study are available from the corresponding author on reasonable request.
